# DAILY ACTIVITY DIVERSITY AND DAILY WORKING MEMORY IN COMMUNITY-DWELLING OLDER ADULTS

**DOI:** 10.1093/geroni/igac059.014

**Published:** 2022-12-20

**Authors:** Minxia Luo, Robert Moulder, Laura Breitfelder, Christina Röcke

**Affiliations:** University of Zurich, Zurich, Zurich, Switzerland; Institute of Cognitive Science, Colorado University Boulder, Boulder, Colorado, United States; University Research Priority Program (URPP) Dynamics Of Healthy Aging, University Of Zurich, Zurich, Zurich, Switzerland; University of Zurich, Dynamics of Healthy Aging & Center for Gerontology, Zurich, Zurich, Switzerland

## Abstract

Research has shown that diverse activity engagement has positive effects on cognitive functioning in older age. However, it is unknown whether the positive effect holds within persons across days and across people. We examined daily within-person association between activity diversity and working memory in older age and effects of potential moderators therein. We examined 16-day smartphone-based ambulatory assessment data from 150 older adults (aged 65+). Participants reported their present activities and completed working memory tasks seven times per day. Within persons, higher daily activity diversity was positively associated with higher daily working memory. Moreover, the prior day’s activity diversity led to that day’s higher working memory, but not vice versa. We did not find any moderating effects of age, education, or fluid and crystallized intelligence. Our results strengthen the evidence on the beneficial effect of activity diversity on cognitive performance. Results are discussed in the context of cognitive reserve theory.

